# Prevalence of reported penicillin allergy and associations with perioperative complications, length of stay, and cost in patients undergoing elective cancer surgery

**DOI:** 10.1017/ash.2023.501

**Published:** 2023-12-13

**Authors:** Nico Christian Grossmann, Yves Kersting, Andres Affentranger, Luca Antonelli, Fabian Joel Aschwanden, Philipp Baumeister, Gerhard Müllner, Marco Rossi, Agostino Mattei, Christian Daniel Fankhauser

**Affiliations:** 1 Department of Urology, Kantonsspital Luzern, Lucerne, Switzerland; 2 Department of Dermatology and Allergology, Kantonsspital Luzern, Lucerne, Switzerland; 3 Department of Infectious Diseases, Kantonsspital Luzern, Lucerne, Switzerland; 4 University of Zurich, Zurich, Switzerland; 5 University of Lucerne, Lucerne, Switzerland

## Abstract

**Objective:**

Up to 10% of patients report penicillin allergy (PA), although only 1% are truly affected by Ig-E-mediated allergies. PA has been associated with worse postoperative outcomes, but studies on the impact of reported PA in cancer patients are lacking, and especially in these multimorbid patients, a non-complicated course is of utmost importance.

**Methods:**

Retrospective analysis of patients undergoing elective oncological surgery at a tertiary reference center. Data on surgical site infections (SSI), postoperative complications (measured by Clavien-Dindo classification and Comprehensive Complication Index (CCI)), hospitalization duration, and treatment costs were collected.

**Results:**

Between 09/2019 and 03/2020, 152 patients were identified. 16/152 patients (11%) reported PA, while 136/152 (89%) did not. There were no differences in age, BMI, Charlson Comorbidity Index, and smoking status between groups (p > 0.4). Perioperative beta-lactam antibiotics were used in 122 (89.7%) and 15 (93.8%) patients without and with reported PA, respectively. SSI and mean numbers of infections occurred non-significantly more often in patients with PA (*p* = 0.2 and *p* = 0.47). The median CCI was significantly higher in PA group (26 vs. 51; *p* = 0.035). The median hospitalization duration and treatment costs were similar between non-PA and PA groups (4 vs 3 days, *p* = 0.8; 16’818 vs 17’444 CHF, *p* = 0.4).

**Conclusions:**

In patients undergoing cancer surgery, reported PA is common. Failure to question the unproven PA may impair perioperative outcomes. For this reason, patient and provider education on which reactions constitute a true allergy would also assist in allergy de-labeling. In addition, skin testing and oral antibiotic challenges can be performed to identify the safe antibiotics and to de-label appropriate patients.

## Introduction

Penicillin allergy is one of the most frequently self-reported allergies in today’s health care with approximately 10% of patients reporting a penicillin allergy.^1^ Nevertheless only 1% of the general population are truly affected by Ig-E-mediated allergies and most lose their sensitivity during follow-up.^2^ Reported penicillin allergies have been shown to be associated with worse patient outcomes. They are associated with surgical site infections, increased prevalence of multidrug-resistant bacteria, increased adverse events, higher mortality, longer hospital stays, and higher treatment costs by the use of alternative antibiotics.^3-6^ Reporting a therefore most likely unjustified penicillin allergy is very common but seems to have negative consequences. Since cancer patients undergoing oncological surgery are usually older, have more comorbidities and other risk factors that can influence a worse perioperative course (e.g. smoking), it is particularly important to know the influence of penicillin allergy in these patients and thus possibly prevent it.

However, none of the numerous studies that investigated the impact of penicillin allergy on perioperative outcomes included cancer patients; hence, there is no evidence in the current literature about the effect in oncologic surgery.

Therefore, the aim of this analysis was to assess the prevalence of reported penicillin allergy and the association with surgical site infections, complications, hospitalization duration, and treatment costs in patients undergoing elective cancer surgery.

## Methods

We retrospectively reviewed all patients who underwent elective oncological surgery at a tertiary reference center between September 2019 (after introduction of a new electronic health record software) and March 2020 (before the COVID wave). We included patients from different specialties including visceral, gynecological, urological, head and neck and thoracic cancers. Exclusion criteria were minor procedures such as surgery under local anesthesia or endoscopic intervention (*n* = 35), procedures without necessary perioperative antibiotic prophylaxis (*n* = 0), unavailability of data on the perioperative antibiotic regimen (*n* = 4), and postoperative follow-up of less than 30 days (*n* = 0). Patients were then divided into two groups, either in the group without reported penicillin allergy (group 1) or in the penicillin allergy group (group 2).

The primary objective of this study was to compare the incidence of surgical site infections, complication rate, and severity between the two groups. Surgical site infection was defined as skin or wound infection or abscess at the surgery site. Complications were recorded within the first 30 days after surgery, and the severity of complications was assessed using the Clavien-Dindo classification (CDC).^7^ CDC grade I were not evaluated, arguing that most of them would not have been reliably entered into the patient’s medical record by clinicians. To summarize complications over time for each procedure, the Comprehensive Complication Index (CCI)^8^ was used. The CCI summarizes complications for each patient in a numerical value between 0 and 100, representing the total burden of complications, whereby 0 represents no complication and 100 represents patient death. The secondary objective of the study was to compare length of hospital stay and total treatment costs between the two groups.

The following characteristics were extracted from the medical chart: patients’ baseline characteristics, reported penicillin allergy, perioperative antibiotics applied, type of surgical procedure, peri- and postoperative infection, infection type, infection site, costs, and length of inpatient stay.

All patients received at least one single-shot intraoperative antibiotic therapy according to the in-house recommendation for perioperative antibiotic prophylaxis. In cases of reported penicillin allergy, the choice of alternative antibiotic therapy was made in adherence to our in-house infectious disease guidelines. These guidelines are formulated based on the broader guidelines pertinent to the region of the operation and consider the local antimicrobial resistance scenarios. The Department of Infectious Diseases is responsible for the regular updating of these guidelines. The postoperative follow-up was not standardized and was performed at the surgeon’s discretion or according to the corresponding oncological guideline.

Fisher’s exact test and Pearson’s chi-squared test were used to assess associations between categorical variables, while Mann–Whitney U-test was used to assess differences in continuous variables between the two surgical techniques. All tests were two-sided, and a *p*-value < 0.05 was considered as statistically significant. All statistical analyses were performed using R (Version 4.0.3, Vienna, Austria, 2020). All patients enrolled had previously signed a general consent, and the study was approved by the local ethics committee (BASEC ID 2020-02389).

## Results

A total of 191 patients were identified. After applying the inclusion and exclusion criteria, 152 patients remained for the final analysis. Of these, 16 (11%) patients reported a penicillin allergy while 136 (89%) did not. Patients median age was 62 (IQR 53, 71) years. Patient characteristics of the total cohort and of the two groups are summarized in Table 1.

**Table 1.** Baseline characteristics of all patients and stratified by reported penicillin allergy

	Total cohort	Reported penicillin allergy	
Characteristics	*n* = 152	No (Group 1), *n* = 136	Yes (Group 2), *n* = 16
**Age**	62 (53, 71)	62 (53, 71)	64 (53, 67)
**BMI**	26.1 (22.9, 29.0)	26.1 (23.1, 29.1)	24.4 (22.4, 27.5)
Missing	56	50	6
**Charlson comorbidity index**	5 (4, 6.5)	5 (4, 6)	4.5 (4, 7.5)
Missing	1	1	0
**Smoking status**			
Never smoked	78 (53%)	67 (52%)	11 (69%)
Former smoker	42 (29%)	38 (29%)	4 (25%)
Current smoker	26 (18%)	25 (19%)	1 (6.2%)
Missing	6	6	0
**Other reported antibiotic allergies**	5 (3.3%)	4 (2.9%)	1 (6.2%)
**Number of reported antibiotic allergies**			
1	19 (95%)	4 (100%)	15 (94%)
2	1 (5.0%)	0 (0%)	1 (6.2%)
**Perioperative antibiotic prophylaxis applied**			
Cefazolin	116 (76%)	102 (75%)	14 (88%)
Cefazolin and Metronidazole	17 (11%)	16 (12%)	1 (6.2%)
Co-Trimoxazole	0 (0%)	0 (0%)	0 (0%)
Clindamycin	2 (1.3%)	1 (0.7%)	1 (6.2%)
Amoxicillin	5 (3.3%)	5 (3.7%)	0 (0%)
Unknown	12 (7.9%)	12 (8.8%)	0 (0%)
**Perioperative beta-lactam antibiotics**	137 (90.1%)	122 (89.7%)	15 (93.8%)

Note. IQR: Interquartile range.

Median (IQR); *n* (%).

Wilcoxon rank sum test; Fisher’s exact test.

Groups did not differ in BMI, Charlson Comorbidity Index, smoking status, other reported antibiotic allergies, number of reported allergies, perioperative antibiotic prophylaxis they received, and the perioperative use of beta-lactam antibiotics (all *p* > 0.2). The types of surgery performed in both groups are listed in Table 2.

**Table 2.** Performed surgeries within the two groups

	No penicillin allergy (Group 1), *n* = 136	Reported penicillin allergy (Group 2), *n* = 16
**Surgery**		
Hysterectomy	10 (7.4%)	1 (6.2%)
Prostatectomy	10 (7.4%)	–
Adnexectomy	2 (1.5%)	–
Cystoprostatectomy	2 (1.5%)	–
Kidney/Ureter/Adrenal Resection	2 (1.5%)	–
Mastectomy	63 (46%)	11 (69%)
Gastrectomy	2 (1.5%)	2 (12%)
HNO MKG tumor resection	15 (11%)	1 (6.2%)
Rectum amputation	3 (2.2%)	–
Wedge resection liver	2 (1.5%)	–
Pancreas resection	4 (2.9%)	–
Lung resection	7 (5.1%)	–
Deep lymph node resection retro/pelvic	4 (2.9%)	1 (6.2%)
Colpectomy	1 (0.7%)	–
Colon resection	5 (3.7%)	–
Omentectomy	1 (0.7%)	–
Laryngopharyngectomy	1 (0.7%)	–
Neck dissection	2 (1.5%)	–

*n* (%).

Table 3 lists the results of the total cohort and two groups after surgical procedures. Surgical site infections occurred more often in group 2 (3 patients, 19%) compared to group 1 (11 patients, 8.1%), but this showed to be non-statistically different (*p* = 0.2). There were no significant differences in the incidence of postoperative urinary tract infection, and none of the patients experienced sepsis. Total complications were the same in both groups with 46 (34%) in group 1 and 6 (38%) in group 2 (*p* = 0.8). There was also an increased mean number of complications in group 2 (1.75) compared to group 1 (0.89), but this was not statistically significantly different (*p* = 0.47). Major complications, which included all with CDC of 3 or higher, were more common in group 2, but this was not found to be statistically significantly different (*p* = 0.2). The CCI was found to be significantly higher in group 2 with 51 (IQR 41, 61), compared to group 1 with 26 (IQR 21, 45) (*p* = 0.035).

**Table 3.** Postoperative outcome characteristics of all patients and stratified by reported penicillin allergy

	Total cohort	Reported penicillin allergy		
Characteristics	*n* = 152	No (Group 1), *n* = 136	Yes (Group 2), *n* = 16	*p*-value
**Overall complications occurred**	52 (34%)	46 (34%)	6 (38%)	0.8
**Number of complications per patient**				0.47
Median (IQR)	0.00 (0.00, 1.00)	0.00 (0.00, 1.00)	0.00 (0.00, 3.00)	
Mean (SD)	1.00 (1.85)	0.89 (1.67)	1.75 (2.96)	
**Highest Clavien-Dindo classification**				0.3
CDC grade II	16 (11%)	16 (12%)	0 (0%)	
CDC grade IIIa	25 (16%)	20 (15%)	5 (31%)	
CDC grade IIIb	8 (5.3%)	7 (5.1%)	1 (6.2%)	
CDC grade IVa	0 (0%)	0 (0%)	0 (0%)	
CDC grade IVb	3 (2.0%)	3 (2.2%)	0 (0%)	
CDC grade V	0 (0%)	0 (0%)	0 (0%)	
**Major complications (≥ Clavien-Dindo III)**	36 (24%)	30 (22%)	6 (38%)	0.2
**Comprehensive Complication Index**	26 (21, 49)	26 (21, 45)	51 (41, 61)	**0.035**
**Surgical site infection**	14 (9.2%)	11 (8.1%)	3 (19%)	0.2
Skin or wound infection	11 (7.2%)	8 (5.9%)	3 (19%)	
Abscess	3 (2.0%)	3 (2.2%)	0 (0%)	
**Urinary tract infection**	4 (2.6%)	4 (2.9%)	0 (0%)	>0.9
**Sepsis**	0 (0%)	0 (0%)	0 (0%)	
**Length of stay** (days)	4 (3, 6)	4 (3, 6)	3 (3, 6)	0.8
**Total treatment costs** (CHF)	17,059 (10,638, 22,579)	16,818 (10,635, 22,534)	17,444 (16,004, 22,599)	0.4
Missing	5	4	1	

Note. IQR: Interquartile range; SD: standard deviation; CDC: Clavien-Dindo classification.

*n* (%); Median (IQR).

Pearson’s chi-squared test; Fisher’s exact test; Wilcoxon rank sum test.

There were no differences in hospitalization duration and total treatment costs. The median hospitalization duration was 4 (IQR 3, 6) days and 3 (IQR 3, 6) days in groups 1 and 2, respectively. The median treatment costs were CHF 16,818 (IQR 10,635, 22,534) and CHF 17,444 (16,004, 22,599) in groups 1 and 2, respectively.

## Discussion

In the present study, we found that patients with reported penicillin allergy who underwent oncologic surgery had a non-significantly higher risk for surgical site infections, complications per patient, and overall complications. A significantly higher CCI was found in patients with penicillin allergy compared to patients without reported penicillin allergy. We found no difference between groups in terms of length of hospital stay and higher treatment costs.

Our results extend the literature describing the association of reported penicillin allergy with postoperative complications. Most of them focused on the effect of penicillin allergy in patients undergoing orthopedic surgery.^5,9-15^ Some others examined this in patients undergoing colorectal and abdominal surgery,^9,16,17^ cardiac interventions,^9^ oral surgery,^18^ or gynecologic surgery.^9^ However, to the best of our knowledge, there is no study that has investigated the impact of a reported penicillin allergy in patients who have undergone cancer surgery.

Most of the studies that investigated the impact of reported penicillin allergy on postoperative outcomes defined the occurrence of surgical site infections as the primary end point and were able to demonstrate a higher incidence in patients with penicillin allergy.^5,9,18^ In a 2017 study by Blumenthal and colleagues, the authors retrospectively examined the impact of reported penicillin allergy on the incidence of surgical site infections in 8,385 patients undergoing hip arthroplasty, knee arthroplasty, hysterectomy, colon surgery, and coronary artery bypass grafting and found reported penicillin allergy in 11% of patients. Moreover, in their descriptive statistics, they could show a higher but not significantly risk for infectious complications in patients with reported penicillin allergy compared those without penicillin allergy, with 3.5% versus 2.6%, respectively (*p* = 0.1). In their multivariable marginal structural model, the effect of reporting penicillin allergy on the development of SSI was fully mediated by receipt of non-beta-lactam antibiotics. The same conclusion was reached by Wilhelm and colleagues, who identified the use of non-beta-lactam antibiotics as perioperative prophylaxis as the sole independent predictor of surgical site infection in their multivariable regression analysis.^19^ The use of beta-lactam antibiotics in 94% of our patients with reported penicillin allergy may thus explain why we did not detect a significant difference in the incidence of infectious complications, as shown in other studies.

However, despite recommendations in most guidelines to use beta-lactam antibiotics as perioperative prophylaxis, the literature suggests that non-beta-lactam antibiotics such as vancomycin, clindamycin, gentamycin, or fluoroquinolones are commonly used in patients with reported penicillin allergy.^3,9^ This is due to the fear of possible cross-reactivity between penicillin and other beta-lactams. However, cephalosporins and other beta-lactams are known to be widely safe to use in patients with established penicillin allergy because the likelihood of immunologic cross-reactivity is very low.^3,20^ Thus, a 2021 systematic review and meta-analysis demonstrated a dual antibiotic allergy to penicillin and cefazolin of 0.6% in patients with reported penicillin allergy and 3% in patients with verified penicillin allergy.^21^ However, the literature recommends preoperative testing of reported penicillin allergy to ensure, if indicated, beta-lactam antibiotic prophylaxis to prevent even the small possibility of a cross-reaction and postoperative infection complications.^22^ In case of rapid assessment of penicillin allergy in cancer patients requiring urgent surgery, the PEN-FAST score is an established rule that accurately identifies patients at low risk of allergy.^23^ We believe that allergy de-labeling is particularly important in cancer patients, as they tend to be older and have more comorbidities, exposing them to an even higher risk of complications.

The CCI is known to be a more sensitive alternative to measure overall morbidity instead of counting the number of complications. It is calculated as the sum of all Clavien-Dindo complications weighted by their severity^8^. Moreover, this continuous complication score has the advantage of reflecting the overall burden of the postoperative course that affects patients’ health and quality of life^24^. In the present study, the influence of reported penicillin allergy on CCI was investigated for the first time, and we recorded a significantly higher CCI in these patients. Fifty percent of the complications recorded in the penicillin allergy group were surgical site infections and more complications were recorded per patient, which explains the higher CCI. Thus, it appears that if an infectious complication occurs, it has a severe downstream effect and add more postoperative complications. This could be explained by the need to manage infection complications with alternative antibiotics, which in turn may promote further adverse postoperative outcomes by additional complications.

Previous studies investigating the impact of reported penicillin allergy on hospitalization duration and treatment costs have reported conflicting results.^5,6,16,25,26^ Our patient groups showed no difference in hospitalization duration and treatment costs, but this should be considered with caution as our cohort consists of patients with different surgical procedures, which in turn are associated with different hospitalization durations and costs and thus may be strong confounders for these end points.

The present study has several limitations. First, the retrospective study design did not include potential confounders for the occurrence of postoperative complications, such as simple or multiple comorbidities. Furthermore, we lack specific data on wound classifications, which could influence surgical site infections and related outcomes. Moreover, it should be noted that patients with different cancer types at different disease stages were included, and that, in addition to the heterogeneity of the surgeries performed, may have an impact on complications, hospitalization duration, and treatment costs. Second, the small single-center cohort allowed only a purely descriptive analysis, so that we recorded an insufficient number of end points, and thus, no analyses with adjustment for possible confounders were possible.

In patients scheduled for elective cancer surgery, reported penicillin allergy is common. Failure to question the unproven antibiotic allergies may impair perioperative outcomes. For this reason, patient and provider education on which reactions constitute a true allergy would also assist in allergy de-labeling. In addition, skin testing and oral antibiotic challenges can be performed to identify the safe antibiotics and to de-label appropriate patients.

## References

[1] Gilissen L , Spriet I , Gilis K , et al. Prevalence of antibiotic allergy labels in a tertiary referral center in Belgium. J Allergy Clin Immunol Pract 2021;9:2415–2425.e8. 33607341 10.1016/j.jaip.2021.01.047

[2] Trubiano JA , Adkinson NF , Phillips EJ. Penicillin allergy is not necessarily forever. JAMA 2017;318:82–83. 28672303 10.1001/jama.2017.6510 PMC5935455

[3] Blumenthal KG , Peter JG , Trubiano JA , et al. Antibiotic allergy. Lancet 2019;393:183–198. 30558872 10.1016/S0140-6736(18)32218-9 PMC6563335

[4] Blumenthal KG , Lu N , Zhang Y , et al. Recorded penicillin allergy and risk of mortality: A population-based matched cohort study. J Gen Intern Med 2019;34:1685–1687. 31011962 10.1007/s11606-019-04991-y PMC6712108

[5] Roebke AJ , Malik AT , Khan SN , et al. Does a reported penicillin allergy affect outcomes following elective posterior lumbar fusions? Int J Spine Surg 2022. 10.14444/8326 PMC9807058 35831066

[6] Pérez-Encinas M , Lorenzo-Martínez S , Losa-García JE , et al. Impact of penicillin allergy label on length of stay and mortality in hospitalized patients through a clinical administrative national dataset. Int Arch Allergy Immunol 2022;183:498–506. 34923488 10.1159/000520644

[7] Dindo D , Demartines N , Clavien PA. Classification of surgical complications: A new proposal with evaluation in a cohort of 6336 patients and results of a survey. Ann Surg 2004;240:205–13. 15273542 10.1097/01.sla.0000133083.54934.ae PMC1360123

[8] Slankamenac K , Graf R , Barkun J , et al. The comprehensive complication index: A novel continuous scale to measure surgical morbidity. Ann Surg 2013;258:1–7. 23728278 10.1097/SLA.0b013e318296c732

[9] Blumenthal KG , Ryan EE , Li Y , et al. The impact of a reported penicillin allergy on surgical site infection risk. Clin Infect Dis 2018;66:329–336. 29361015 10.1093/cid/cix794 PMC5850334

[10] Lee OC , Cheng DC , Paul JL , et al. Economic burden of patient-reported penicillin allergy on total hip and total knee arthroplasty. J Arthroplasty 2021;36:3067–3072. 34053750 10.1016/j.arth.2021.04.032

[11] Otero JE , Graves CM , Gao Y , et al. Patient-reported allergies predict worse outcomes after hip and knee arthroplasty: Results from a prospective cohort study. J Arthroplasty 2016;31:2746–2749. 27600302 10.1016/j.arth.2016.07.040

[12] Raso J , Kamalapathy PN , Puvanesarajah V , et al. Penicillin allergy in spine surgery: Increased rates of sepsis, emergency room visits, and readmission. World Neurosurg 2022;162:e91–e98. 35227923 10.1016/j.wneu.2022.02.079

[13] Spangehl M. Preoperative prophylactic antibiotics in total hip and knee arthroplasty: What, when, and how. J Arthroplasty 2022;37:1432–1434. 35051610 10.1016/j.arth.2022.01.019

[14] Stone AH , Kelmer G , MacDonald JH , et al. The impact of patient-reported penicillin allergy on risk for surgical site infection in total joint arthroplasty. J Am Acad Orthop Surg 2019;27:854–860. 30829986 10.5435/JAAOS-D-18-00709

[15] Wu VJ , Iloanya MC , Sanchez FL , et al. Is patient-reported penicillin allergy independently associated with increased risk of prosthetic joint infection after total joint arthroplasty of the hip, knee, and shoulder? Clin Orthop Relat Res 2020;478:2699–2709, 33027190 10.1097/CORR.0000000000001497 PMC7899399

[16] Khan A , Wolford DD , Ogola GO , et al. Impact of patient-reported penicillin allergy on antibiotic prophylaxis and surgical site infection among patients undergoing colorectal surgery. Dis Colon Rectum 2022;65:1397–1404. 34856589 10.1097/DCR.0000000000002190

[17] Schlosser KA , Maloney SR , Horton JM , et al. The association of penicillin allergy with outcomes after open ventral hernia repair. Surg Endosc 2020;34:4148–4156. 32016513 10.1007/s00464-019-07183-1

[18] Roistacher DM , Heller JA , Ferraro NF , et al. Is penicillin allergy a risk factor for surgical site infection after oral and maxillofacial surgery? J Oral Maxillofac Surg 2022;80:93–100. 34547269 10.1016/j.joms.2021.08.147

[19] Wilhelm NB , Bonsall TJ , Miller CL . The effect of beta-lactam allergy status on the rate of surgical site infections: a retrospective cohort study. Ann Surg 2022;275:208–212. 32502079 10.1097/SLA.0000000000003949

[20] Macy E. Penicillin and beta-lactam allergy: Epidemiology and diagnosis. Curr Allergy Asthma Rep 2014;14:476. 25216741 10.1007/s11882-014-0476-y

[21] Sousa-Pinto B , Blumenthal KG , Courtney L , et al. Assessment of the frequency of dual allergy to penicillins and cefazolin: A systematic review and meta-analysis. JAMA Surg 2021;156:e210021. 33729459 10.1001/jamasurg.2021.0021 PMC7970387

[22] Reilly CA , Backer G , Basta D , et al. The effect of preoperative penicillin allergy testing on perioperative non-beta-lactam antibiotic use: A systematic review and meta-analysis. Allergy Asthma Proc 2018;39:420–429. 30401320 10.2500/aap.2018.39.4178

[23] Trubiano JA , Vogrin S , Chua KYL , et al. Development and validation of a penicillin allergy clinical decision rule. JAMA Intern Med 2020;180:745–752. 32176248 10.1001/jamainternmed.2020.0403 PMC7076536

[24] Slankamenac K , Nederlof N , Pessaux P , et al. The comprehensive complication index: A novel and more sensitive endpoint for assessing outcome and reducing sample size in randomized controlled trials. Ann Surg 2014;260:757–62. 25379846 10.1097/SLA.0000000000000948

[25] Bhathal S , Joseph E , Nailor MD , et al. Adherence and outcomes of a surgical prophylaxis guideline promoting cephalosporin use among patients with penicillin allergy. Surgery 2022. 10.1016/j.surg.2022.01.020 35183368

[26] Sacco KA , Bates A , Brigham TJ , et al. Clinical outcomes following inpatient penicillin allergy testing: a systematic review and meta-analysis. Allergy 2017;72:1288–1296. 28370003 10.1111/all.13168

